# X-Ray Solution Scattering Study of Four *Escherichia coli* Enzymes Involved in Stationary-Phase Metabolism

**DOI:** 10.1371/journal.pone.0156105

**Published:** 2016-05-26

**Authors:** Liubov A. Dadinova, Eleonora V. Shtykova, Petr V. Konarev, Elena V. Rodina, Natalia E. Snalina, Natalia N. Vorobyeva, Svetlana A. Kurilova, Tatyana I. Nazarova, Cy M. Jeffries, Dmitri I. Svergun

**Affiliations:** 1 A.V. Shubnikov Institute of Crystallography of Federal Scientific Research Centre “Crystallography and Photonics” of Russian Academy of Sciences, Moscow, Russia; 2 M.V. Lomonosov Moscow State University, Physics Department, Moscow, Russia; 3 A.N. Belozersky Institute of Physico-Chemical Biology, Moscow State University, Moscow, Russia; 4 M.V. Lomonosov Moscow State University, Chemistry Department, Moscow, Russia; 5 EMBL, Hamburg Outstation, c/o DESY, Hamburg, Germany; University of Canterbury, NEW ZEALAND

## Abstract

The structural analyses of four metabolic enzymes that maintain and regulate the stationary growth phase of *Escherichia coli* have been performed primarily drawing on the results obtained from solution small angle X-ray scattering (SAXS) and other structural techniques. The proteins are (i) class I fructose-1,6-bisphosphate aldolase (FbaB); (ii) inorganic pyrophosphatase (PPase); (iii) 5-keto-4-deoxyuronate isomerase (KduI); and (iv) glutamate decarboxylase (GadA). The enzyme FbaB, that until now had an unknown structure, is predicted to fold into a TIM-barrel motif that form globular protomers which SAXS experiments show associate into decameric assemblies. In agreement with previously reported crystal structures, PPase forms hexamers in solution that are similar to the previously reported X-ray crystal structure. Both KduI and GadA that are responsible for carbohydrate (pectin) metabolism and acid stress responses, respectively, form polydisperse mixtures consisting of different oligomeric states. Overall the SAXS experiments yield additional insights into shape and organization of these metabolic enzymes and further demonstrate the utility of hybrid methods, i.e., solution SAXS combined with X-ray crystallography, bioinformatics and predictive 3D-structural modeling, as tools to enrich structural studies. The results highlight the structural complexity that the protein components of metabolic networks may adopt which cannot be fully captured using individual structural biology techniques.

## Introduction

The transition of bacterial cells to the stationary phase of growth may result in lowered metabolic activity combined with an increase in resistance to stress effects caused by nutrient deprivation [1]. Many cellular processes undergo changes during stationary phase or are induced/activated in order to provide protective mechanisms against unfavorable environmental conditions [2]. Understanding the regulatory mechanisms of particular proteins involved in metabolism as well as the overall structural characteristics of these proteins are important for understanding the complexity of cell metabolic regulation and stress responses [2]. This article focuses on a set of four of *Escherichia coli* enzymes believed to participate in metabolic regulation in the stationary phase cells and synthesizes the results obtained from hybrid structural biology methods to provide additional insights into the organization of these proteins in solution. The proteins include a new type of fructose-1,6-bisphosphate aldolase (FbaB), inorganic pyrophosphatase (PPase), 5-keto 4-deoxyuronate isomerase (KduI) and glutamate decarboxylase (GadA).

Fructose-1,6-bisphosphate aldolase (Fba) is a key enzyme involved in glycolysis, gluconeogenesis, and fructose metabolism [3, 4]. In eukaryotes Fba also plays a variety of non-enzymatic functions, mediating interactions between different proteins [5–7]. There are two unrelated classes of Fba that differ in their catalytic and structural properties. Class I aldolases are primarily expressed in higher eukaryotes and archaea [8, 9], while Class II aldolases are found in eubacteria and some eukaryotes [10, 11]. Several bacteria, including *E*. *coli*, may express Fba of both classes [12–14]. It has been shown that in *E*. *coli* Class II Fba (coded by the gene *fbaA*) represents a major class of glycolytic activity enzymes, while Class I Fba (coded by *fbaB*) is a minor enzyme expressed in bacteria grown on non-sugar carbon sources [12, 15]. The role of Class I Fba, herein referred to as FbaB, is poorly understood and its structure is unknown, but evidence suggests that the enzyme important for the stress adaptation of bacteria, including dehydration, sugar starvation, and osmotic regulation [12–16]. Given the low amino acid sequence homology (26%) of bacterial FbaB to the closest homolog from archaea and eukaryotes [17], the FbaB from *E*. *coli* described here may represent a member of a new structural family of these aldolases. A considerable part of the present work focuses on the predictive structural modeling of this protein combined with experimental SAXS to study the structure and global organization of enzyme in solution.

The second enzyme investigated in this study, PPase, plays an important role in cell energy metabolism and is involved in the biosynthesis of proteins and nucleic acids [18]. KduI is a sugar isomerase that is also present in the soil bacteria *Erwinia chrisantemii*, where it is known to be involved in the degradation of polysaccharide pectin [19]. The functional role of this enzyme in *E*. *coli* is still unclear as *E*. *coli* lacks additional genes for pectin metabolism [20]. GadA catalyzes the decarboxylation of glutamate to γ-aminobutyrate and is involved in bacterial acid-stress response [21, 22].

To date, the high resolution X-ray crystal structures of PPase, KduI and GadA have been determined and are available in the Protein Data Bank (PDB entries 2AUU [23], 1XRU [24] and 1XEY [22], respectively). However, the overall organization of these enzymes in solution, in particular whether the crystallographic oligomer states are preserved, has not been extensively investigated. The global solution state(s) of these enzymes may differ from what is observed in a crystal matrix when the influences of crystallographic packing forces are removed in solution. Consequently, the results obtained from solution investigations—for example using SAXS—may provide new structural details and information that more adequately describes the natural condition of these proteins *in vivo*. Here, we employ a hybrid approach that utilizes SAXS in combination with predictive 3D-structural modeling (for FbaB) and the previously reported results from X-ray crystallography (PPase, KduI and GadA) to detail the structures and states of these enzymes in solution. SAXS is well suited to analyze the structural organization of multidomain and flexible proteins [25, 26] and allows for the rigid body modeling of the quaternary structure from subunits with fully or partially known atomic structures [27–30]. The results provide a platform for future investigations into the role of these proteins in the mechanisms of cell adaptation to stress.

## Materials and Methods

### Protein expression and purification, size exclusion chromatography and analytical ultracentrifugation

Fructose-1,6-bisphosphate aldolase (FbaB), inorganic pyrophosphatase (PPase), 5-keto 4-deoxyuronate isomerase (KduI) and glutamate decarboxylase (GadA) were cloned and expressed in *Eschericia coli*, and purified as previously described [31].

Size-exclusion chromatography (SEC) was performed using Superdex 200 10/300 GL column (GE Healthcare) pre-equilibrated with at either high- or low-pH buffers: 50 mM Tris-HCl, 10 mM NaCl, 1 mM DTT pH 7.5 or; 100 mM Na-acetate, 10 mM NaCl, 1 mM DTT pH 4.6. In all instances the protein load concentration was 5 mg/ml. Molecular mass estimates of the eluting sample components were calculated relative to the migration of protein standards through the SEC column in the respective high- or low-pH buffers. The SEC column calibration was performed using a protein molecular mass standard kit for gel filtration chromatography (GE Healthcare) that contained 8 globular proteins with molecular masses from 1.35 to 669 kDa.

Analytical ultracentrifugation (AU) was performed at 20°C in a Spinco E instrument (Beckman Instruments) equipped with a computerized data collection unit with scanning at 280 nm. The sedimentation velocity of each protein sample was measured in triplicate at 260 000 × *g*, and sedimentation coefficient (*s*_20,w_) and molecular mass were calculated with the in-built SedFit program. Samples were analyzed at 0.4 mg/ml in either 100 mM, Tris-HCl, 10 mM NaCl, 1 mM DTT pH 7.5 or 100 mM, Na-acetate, 10 mM NaCl, 1 mM DTT pH 4.6.

### Homology structural modeling of the FbaB monomer

The amino acid sequence of Class I fructose bisphosphate aldolase, FbaB, from *E*. *coli* was retrieved from UniProt database (A0A037Y4V8) [32]. The protein contains 350 amino acids with a calculated monomeric molecular weight of 38.1 kDa. The 2D secondary structure prediction of the monomer was performed using PsiPred server [33], and the 3D model was generated by I-Tasser [34]. This online resource uses a multiple threading algorithm to identify structurally similar templates against a PDB library of chain fragments. The full-length structure models are then constructed by iterative simulations, and ranked based on their C-score (quantitative measure of the confidence), TM score (quantitative assessment of protein structural similarity) and RMSD. The structure validation and quality control was done by Procheck [35] and WhatCheck module on WhatIf server [36].

### Scattering experiments and data analysis

Synchrotron SAXS measurements were performed at the European Molecular Biology Laboratory (EMBL) on the EMBL-P12 BioSAXS beamline at the PETRAIII storage ring (DESY, Hamburg) equipped with a robotic sample changer and a 2D photon counting pixel X-ray detector Pilatus 2M (DECTRIS, Switzerland). The scattering intensity, *I*(*s*), was recorded in the range of the momentum transfer 0.08 < *s* < 4.5 nm^-1^, where *s =* (4πsin*θ*)/λ, 2*θ* is the scattering angle, and λ *=* 0.124 nm, the X-ray wavelength [37]. The measurements were carried out in a standard 50 mM Tris, pH 7.5, buffer at 10°C using continuous flow operation over a total exposure time of 1 s divided into 20 x 50 ms individual frames to monitor for potential radiation damage (no radiation effects were detected) [38]. The data were corrected for the solvent scattering and processed using standard procedures [39]. To account for the interparticle interactions, solutions of PPase, FbaB, GadA and KduI at four proteins concentrations in the range of 1.4–10.8 mg/ml were measured. For GadA, the samples were stored in 100 mM Na-acetate, 10 mM NaCl, 1 mM DTT pH 4.6 prior to raising the pH to 7.5 in the standard Tris buffer immediately before the SAXS measurements. An additional data set ‘GadA low-salt’ was measured from a sample of GadA at 5 mg/ml derived from a stock solution at a lower ionic strength without the addition of 10 mM NaCl to the supporting solvent.

The values of the total forward scattering at zero angle, *I*(0), and radii of gyration, *R*_*g*_, were calculated from the experimental SAXS profiles using the Guinier approximation, which is valid in the range of (*sR*_*g*_) approximately < 1.3 [40]. These parameters and the maximum particle dimension, *D*_*max*_, were also computed from the distance distribution function, *p*(*r*), using program GNOM [41]. The molecular masses (MM) of each sample were calculated from the SAXS data using the value of *I*(0) (MM_*I*(0)_) combined with protein concentration relative to a bovine serum albumin standard [42] as well as from the concentration-independent excluded Porod volume (MM_Porod_) [43]. The latter was determined given that the empirical ratio between the Porod volume (V_*p*_) and MM of a protein is approximately 1.7 [27, 44]. The experimentally determined MM were compared to the expected MM_aa_ calculated from the amino acid sequences of the monomeric forms of the proteins multipled by the protomer stoichiometry observed in previously determined X-ray crystal structure homologues. This comparison was used to assess the oligomerisation state of each enzyme in solution. The MM_aa_ was calculated using [44] applying respective multiplication factors to the monomeric molecular mass: PPase, 6; FbaB, 10; GadA, 6 and; KduI, 6.

### *Ab initio* bead modeling and rigid body modeling against the SAXS data

The low-resolution shapes of FbaB and PPase were reconstructed *ab initio* using the program DAMMIN [45] that generates 3D dummy atom (bead) model spatial representations of a particle. Starting from a random assembly, the program utilizes a simulated annealing (SA) algorithm to build models fitting the experimental data *I*_exp_(*s*) with a minimum discrepancy

$$\chi^{2}=\frac{1}{N-1}\Sigma_{j=1}^{N}{\left[\frac{I_{exp}\left(s_{j}\right)-cI_{calc}\left(s_{j}\right)}{\sigma \left(s_{j}\right)}\right]}^{2}$$ (1)

i.e., the reduced *χ*^2^ test, where *N* is the number of experimental points, *c* is a scaling factor and *I*_calc_(*s*_*j*_) and *σ*(*s*_*j*_) are the calculated intensity from the model and the experimental error on the intensities at the momentum transfer *s*_*j*_, respectively. Repetitive reconstructions were performed (not less than 10 per SAXS experiment) and the models were further analyzed using the programs SUPCOMB [46] and DAMAVER [47] to identify the most typical models representing the global shape of FbaB or PPase in solution (applying P52 and P32 symmetries, respectively). Comparison of the models was performed using the normalized spatial discrepancy, NSD. Generally an average NSD ≤ 1 indicates that the models are similar, while the NSD significantly exceeding one suggests large variations between the individual reconstructions [46]. The Correlation Map method was also used to assess the quality of the model fits (as an alternative to the reduced *χ*^2^ test) [48].

Rigid-body modeling was performed using a suite of programs [27] where either homology models (in the case of FbaB) or the available high-resolution X-ray crystal structures of the enzymes (PPase, KduI and GadA) were used as inputs in the refinement calculations. Rigid body modeling using SASREF [28] in P32 symmetry was applied to refine the relative positions of the individual protomers of PPase to achieve the best fit to the experimental data. SASREFMX was used to perform rigid-body modeling of GadA, again in P32 symmetry, taking into account the disassociation of the hexamer into dimers. The program CORAL [27] was employed to refine the spatial arrangement of the FbaB protomers of the protein’s quaternary structure using the homology model of the monomer developed in this study. CORAL modeling was performed in P52 symmetry whereby the N-terminal extension to the folded core of the homology model (amino acids 1–36) were represented as a string of amino acids. Where applicable, CRYSOL [49] was used to evaluate the fits to the data of the final SASREF and CORAL models as well as to assess the fits against the SAXS data of the previously reported crystal structures for PPase, KduI and GadA. Finally, to determine the number of the components that best describe the state of KduI or GadA in solution, e.g., monomers, dimers, hexamers, etc, the program OLIGOMER [50] was used. For a system containing a distinct number of components, the scattering profile can be described as the volume-fraction weighted linear combination of each component in the mixture, i.e.,

$$I\left(s\right)=\Sigma_{i=1}^{M}\left(v_{i}I_{i}\left(s\right)\right),$$ (2)

where ν_i_ and *I*_*i*_(*s*) are the volume fraction and the scattering intensity from the *i-*th component, respectively. For KduI, the scattering from the different oligomeric components derived from the hexameric crystal structure were computed using CRYSOL and entered into Eq (2) as implemented in OLIGOMER to find the mixture of components that best describe the experimental data. For GadA, those hexameric and dimeric models derived from SASREFMX refinement were also assessed using OLIGOMER to evaluate the final fit and volume fraction estimates of the GadA components that best describe the experimental SAXS data.

## Results and Discussion

### SAXS study of FbaB, PPase, KduI and GadA

**Fig 1:**
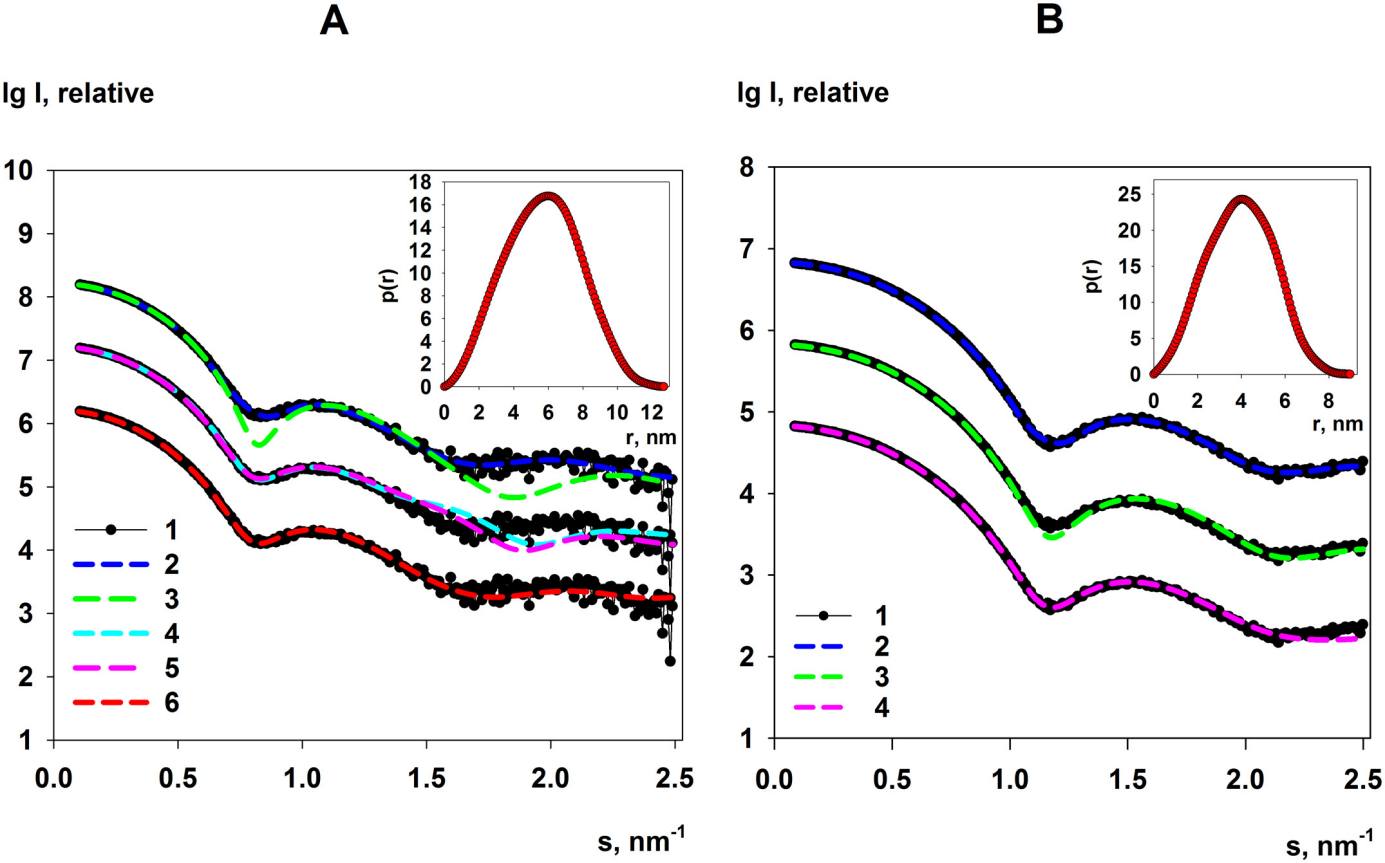
Scattering data and model fits for FbaB and PPase in solution. **A. FbaB:** (1) The experimental FbaB SAXS data at 9.3 mg/ml. (2) The fit to the SAXS data of the *ab initio* FbaB DAMMIN model displayed in Fig 6. (3) and (4) SASREF rigid-body model fits of FbaB octamers and decamers, respectively. (5) The fit to the data of an archaeal FbaB decamer (PDB: 1OJX). (6) The FbaB CORAL model fit to the data. **B. PPase:** (1) The SAXS profile of the final merged SAXS data set and respective fit (2) to the data of the PPase *ab initio* DAMMIN model displayed in Fig 6C. (3) Shows the fit to the data of the calculated scattering from the X-ray crystal structure of the PPase hexamer and (4) the hexamer modelled against the SAXS data using SASREF as shown in Fig 6B and 6C. Note: for visualization purposes, the identical SAXS profile for either FbaB or PPase are presented in triplicate and shifted on the *I*(*s*) axis. *Inserts*: the corresponding distance distribution functions *p*(*r*) computed by GNOM for FbaB and PPase.

**Fig 2:**
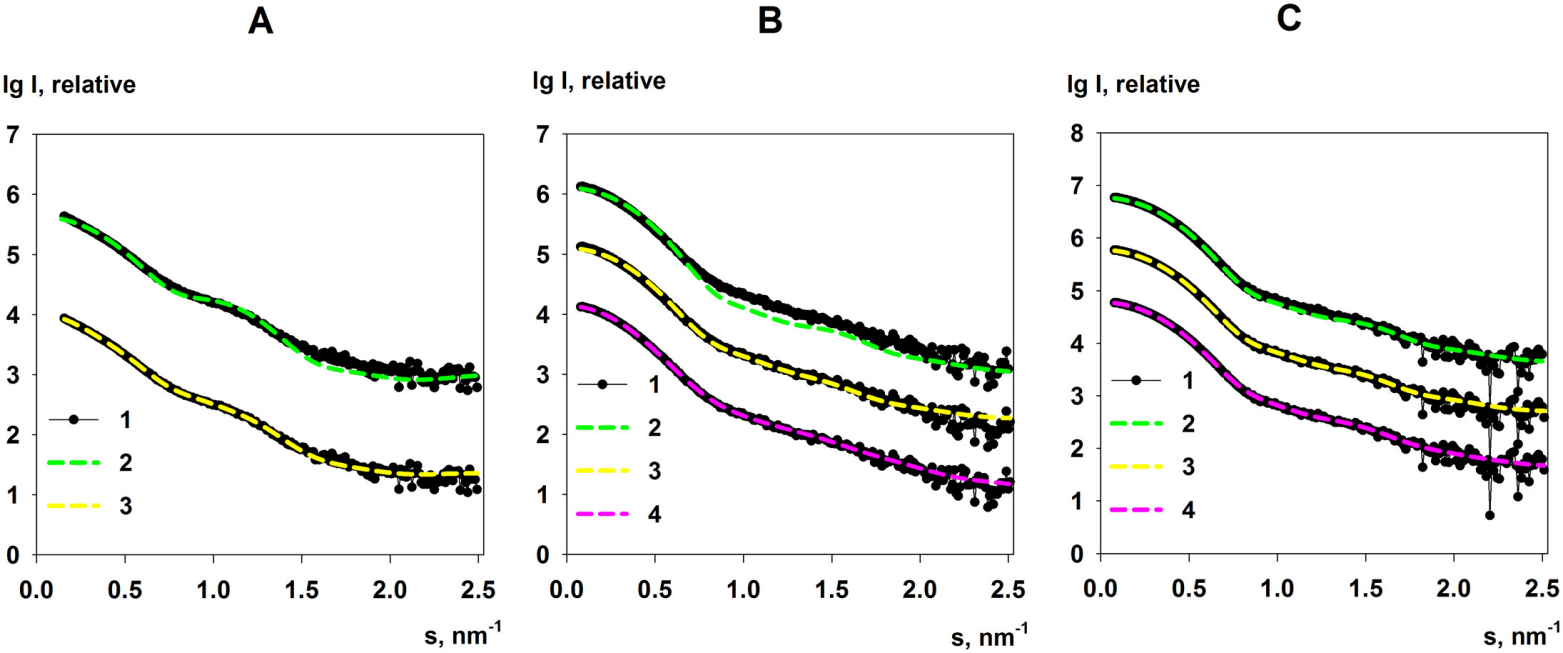
Scattering data from KduI (A) and GadA (B) and GadA low-salt (C). In all panels, (1) represents experimental data and (2) the scattering-fit computed from the respective hexameric X-ray crystal structures of either KduI or GadA. OLIGOMER fits to the data for the respective mixtures are also displayed. For KduI, curve (3)—panel A—shows the fit of the hexamer, dimer and dodecamer mixture of the models presented in Fig 7. For the GadA and GadA low-salt samples, curve (3) shows the OLIGOMER fit of the crystallographic hexamer and all associated crystallographic dimers, while curve (4) shows the best fitting hexamer-dimer mixtures modelled using SASREFMX as displayed in Fig 8A and 8B, respectively. Note: for visualization purposes, the identical SAXS profile for KduI (4 mg/ml), GadA (8.5 mg/ml) and GadA low-salt (5 mg/ml) have been duplicated and shifted on the *I*(*s*) axis.

The processed experimental scattering patterns from FbaB, PPase, KduI and GadA are displayed in Figs 1 and 2. All data and models have been deposited and are available at the Small-angle Scattering Biological Database (SASBDB; www.sasbdb.org [51]) under the accession codes SASDBZ2 (FbaB), SASDBY2 (PPase), SASDB23 (KduI), SASDB33 (GadA) and SASDBS4 (GadA at low-salt). Concentration dependent effects on the measured scattering intensities were not observed for FbaB, KduI and GadA samples in the standard Tris buffer spanning 1.4–10.9 mg/ml (S1 Fig), whereas slight repulsive interparticle interference are noted at low-*s* in the SAXS data for PPase when the sample concentration is increased from 2.8–10.9 mg/ml, specifically for *s* < 0.54 nm^-1^. As the SAXS data measured from all of the PPase samples are statistically similar for higher values of *s*, (*s* > 0.54 nm^-1^, Correlation Map *p* = 0.11–1.0 [48]), irrespective of concentration, the low-*s* SAXS data recorded from the 2.8 mg/ml sample were merged with the *s* > 0.54 nm^-1^ intensities from the highest concentration sample (10.9 mg/ml) using PRIMUS [50] to produce the final PPase SAXS profile.

**Fig 3:**
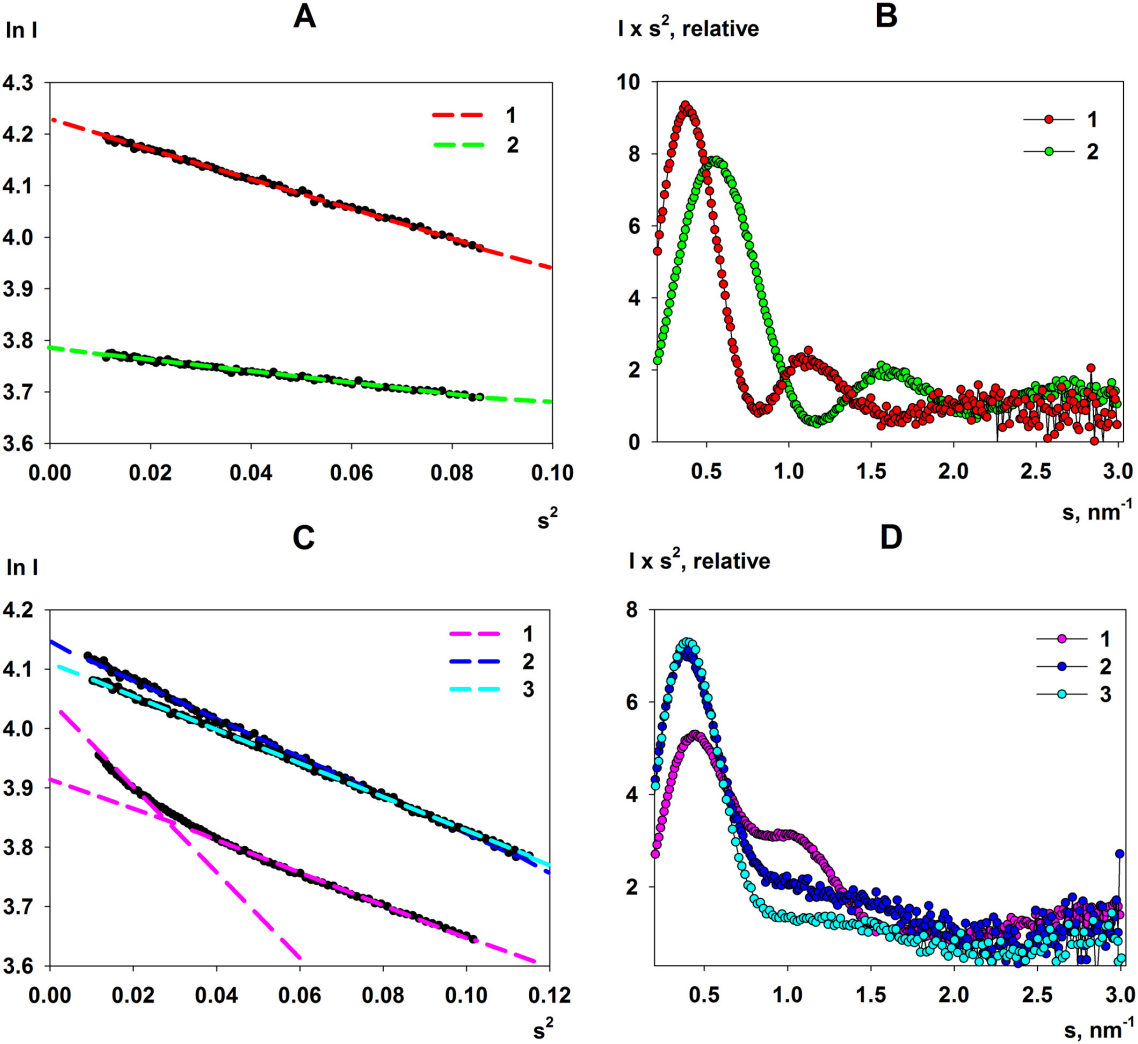
Guinier and Kratky plots for PPase, FbaB, KduI and GadA. **A.** Guinier plots for (1) FbaB and (2) PPase. **B.** The corresponding Kratky plots for FbaB (1) and PPase (2). **C.** Guinier plots for KduI (1) showing a double-slope Guinier region (corresponding to *R*_*g*_ = 6.2 nm and *R*_*g*_ = 4.5 nm). Plots (2) and (3) show the results obtained from the GadA and GadA low-salt samples. **D.** The corresponding Kratky plots of KduI (1), GadA (2) and GadA low-salt (3).

The scattering data from FbaB, PPase and GadA display linear ranges in the Guinier plots determined from the SAXS data while, in contrast, the Guinier plot of KduI is non-linear (Fig 3). Indeed, it is possible to define two predominant regions within the Guinier plot of KduI (*s*^2^, 0.01–0.035 and 0.04–0.11 nm^-2^) from which two values of *R*_g_ can be approximated (6.2 nm and 4.5 nm) suggesting that KduI may exist as a mixture high- and low-MM oligomers in solution. In addition, the SAXS data for GadA changes dramatically on changing the ionic strength (S2 Fig). When SAXS data are recorded from a sample without NaCl present, the *R*_*g*_ decreases from 4.8 nm to 4.4 nm, while the *I*(0) and average MM estimate increases, suggesting significant structural rearrangements occur in the protein in response to a change in sample environment (Table 1; Fig 2B and 2C and see below). The corresponding Kratky plots for all of the proteins used in this study are displayed in Fig 3B and 3D that indicate FbaB, PPase, KduI and GadA, likely adopt well-folded structures in solution. The structural parameters, *R*_*g*_, *D*_*max*_, V_*p*_ and MM estimates extracted from the SAXS data are reported in Table 1.

**Table 1:** Overall structural parameters of the proteins.^a^

Sample	*R*_*g*_, nm	*R*_*g cryst*_, nm	V_*p*_, nm^3^	*D*_*max*_, nm	MM_*I*(0)_, kDa	MM_aa_, kDa	MM_Porod_, kDa
**PPase**	3.0±0.1	3.0	166±20	9.0±0.5	130±10	117	104±10
**FbaB**	4.4±0.1	4.4^b^	570±30	12.7±0.6	340±20	381	335±15
**KduI**	6.2±0.1 4.5±0.1^c^	3.9	310±20	-	183±10	187	182±10
**GadA**	4.8±0.1	4.2	430±20	-	249±15	316	252±15
**GadA low-salt**	4.4±0.1	4.2	450±20	-	260±15	316	265±15

^**a**^Notations: *R*_*g*_, radius of gyration from Guinier; *R*_*g cryst*_, radius of gyration calculated from the available X-ray crystal structures; V_*p*_, Porod volume estimate; *D*_*max*_, maximum particle dimension; MM_*I*(0)_, molecular mass from *I*(0); MM_aa_, calculated oligomeric molecular mass from the amino acid sequence (PPase, KduI, GadA = hexamer; FbaB = decamer); MM_Porod_, molecular mass from Porod volume. The associated errors for *R*_*g*_ and MM_*I*(0)_ represent standard deviations directly evaluated by the error propagation from the experimental data. For V_*p*_, MM_Porod_ and *D*_*max*_, the deviations were assessed by repetitive runs by varying the analysis parameters.

^**b**^*R*_*g cryst*_ for the FbaB PDB entry 1OJX.

^**c**^The *R*_*g*_ determined for KduI were estimated from two slopes observed in the Guinier plot at low angles (*s*^2^, 0.01–0.035 and 0.04–0.11 nm^-2^).

### Modeling the tertiary structure of the FbaB monomer

At present, the high resolution X-ray crystal structure of Class I fructose bisphosphate aldolase from *E*. *coli* has not been determined so we used a combination of well-established secondary and tertiary structure modeling programs to develop an atomistic representation of the FbaB monomer. The amino acid sequence of FbaB has a distant homology to other Class I Fbas from archaea and eukaryotes (e.g., it has 26% identity to the sequence of Fba from *Thermoproteus tenax*). The prediction of the secondary structure of FbaB using PsiPred [33] showed an alternating pattern between α-helices and β-strands along the length of sequence in common with the triose-phosphate isomerase (TIM)-barrel fold. All known Class I aldolases contain the TIM-barrel motif, often with the inclusion of additional secondary structural extensions at the C- or N-termini and/or between the α/β core elements of the barrel (S3 Fig). The *E*. *coli* FbaB studied here has a predicted additional α-helix extension to the TIM-barrel motif that spans the N-terminal region of the protein (amino acids 1–40).

**Fig 4:**
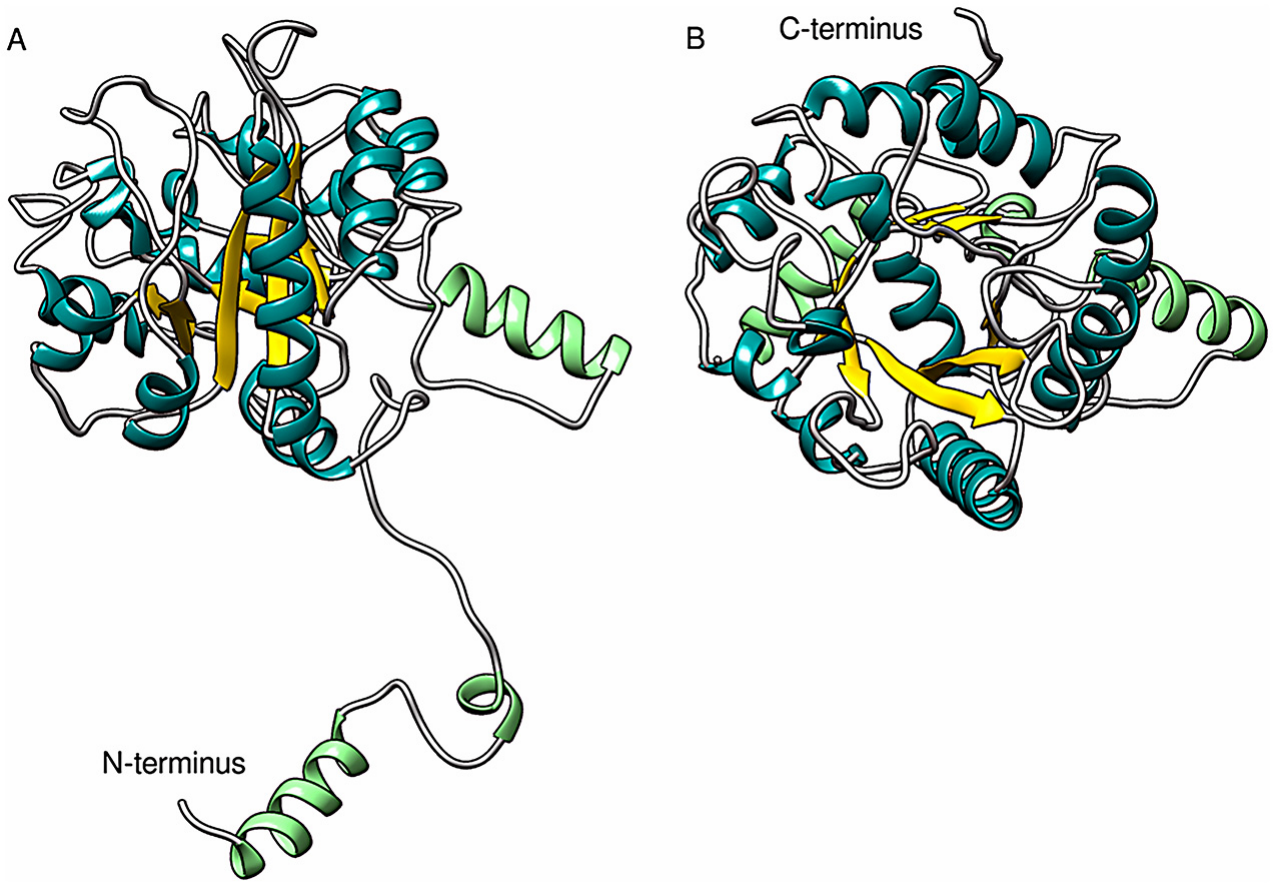
Overall fold of Class I Fba from *E*. *coli* as predicted using secondary and tertiary structure modeling. **A.** A profile view of FbaB Model 1 and; **B.** A top view along the axis of the TIM-barrel fold. The β-strands and α-helices of the TIM barrel are colored gold and dark cyan, respectively. Light green α-helices are not involved in forming the TIM-barrel core motif.

The tertiary structure of FbaB monomer was modeled using I-Tasser [34]. The five best-ranked models were almost identical in their TIM-barrel folds and differed only by the conformations of their N-terminal regions and the region spanning amino acids 253–282 that are predicted to occur at the surface of the protein. The most significant difference between the models was observed at the N-terminus, where a chain fragment 1–40 either contained an α-helix of varied length, or was predicted to be disordered with a variety of conformations. The best predicted model had a C-score of -1.07, estimated TM-score 0.758±0.14, and estimated RMSD 0.89±0.46 nm. When comparing the predicted structure of FbaB to ten of the closest structural analogs in the PDB, three fructose bisphosphate aldolase homologues were identified (PDB: 1OK6, 3MMT, 3BV4) along with four tagatose bisphosphate aldolases (PDB: 3MHG, 3MYO, 3KAO, 3IV3), one 2-amino-3,7-dideoxy-D-threo-hept-6-ulosonic acid synthase (PDB: 3GND) and one protein with unknown function (PDB: 2QJG). The TM-score against the best PDB analog was 0.758 (a fructose bisphosphate aldolase; PDB: 4MOZ) (S1 Table). All of these proteins belong to the Class I aldolase structural superfamily; this fact, together with the high values of the C- and TM-scores, points to a high confidence in the prediction of the 3D structure of the FbaB monomer. The geometry of the selected Model 1 (Fig 4) was further was validated by Procheck [35] and WhatIf [36]. The resulting Ramachandran revealed that over 80% of the amino acids fall in the preferred ϕ/ψ peptide bond angle regions and the model contained only 2% outliers in disallowed regions. The overall geometry and packing validation parameters calculated by WhatIf corresponded to a good-quality model. In Fig 4, the β-strands and α-helices of the TIM barrel of the final FbaB model are shown in gold and dark cyan, respectively, while the secondary structure elements not involved in forming the core motif are shown in green.

**Fig 5:**
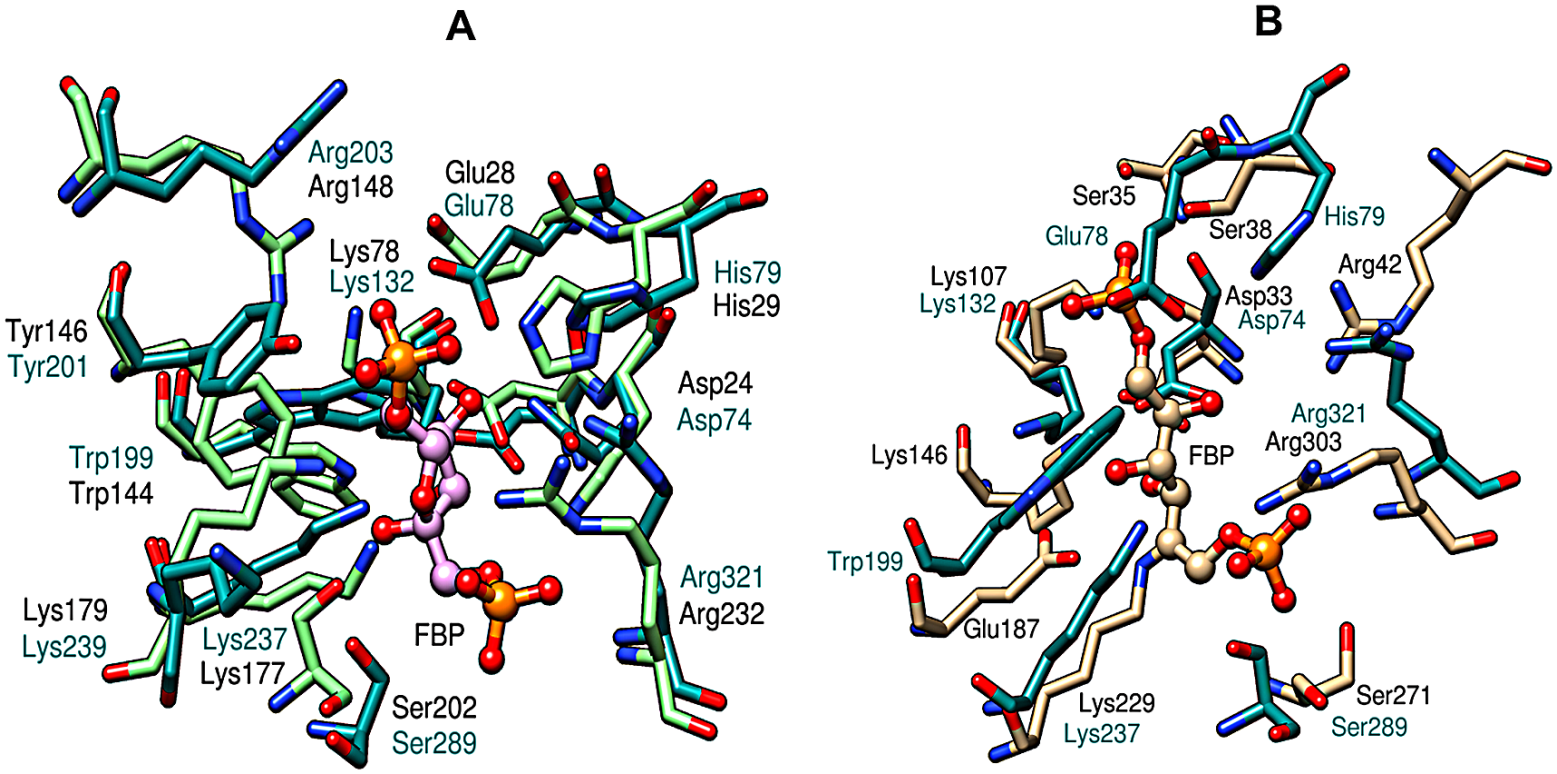
Superposition of the active site residues of Class I Fba. The predicted active site topology of the *E*. *coli* enzyme (derived from I-Tasser, Model 1) is shown in dark green on both figure panels. **A.** Spatial alignment of *E*. *coli* FbaB and the X-ray crystal structure of FbaB from *Thermoproteus tenax* (PDB: 1W8R [52]). **B.** The alignment between *E*. *coli* FbaB relative to *Oryctolagus cuniculus* FbAB (PDB: 1ZAI [9]). The substrate fructose-1,6-bisphosphate (FBP), taken from the 1W8R crystal structure, is represented as a ball-and-stick in panel A. The amino acid numbers of the corresponding proteins are given in black for the X-ray crystal structures and in green for the I-Tasser *E*.*coli* model.

The validity of the predicted structure of FbaB is additionally supported by an agreement between the predicted positions of the active site amino acids of the *E*. *coli* model and the corresponding active site structure of archaeal Fba from *Thermoproteus tenax* [52] (PDB: 1W8R). It can be seen in Fig 5A that even though both enzymes share low overall amino acid sequence homology (26%) most of the active site amino acids of the *E*. *coli* enzyme are spatially located within a similar hydrogen bonding distances relative to the bound fructose-1,6-bisphosphate (FBP) substrate of archaeal Fba. This includes the catalytic amino acids Tyr146 (Tyr201 in *E*. *coli*), Asp24 (74), and Lys177 (237) that form a Shiff base to FBP. On the other hand, the superposition of the predicted *E*. *coli* FbaB to the crystal structure of eukaryotic Fba (*Oryctolagus cuniculus*, PDB: 1ZAI [9]) does not reveal spatial similarity on comparing the amino acids that construct the enzyme active sites (Fig 5B). For example, some of the catalytic residues in eukaryotic Fba, e.g., Glu187 and C-terminal Tyr364, do not find structural equivalents in the *E*. *coli* enzyme. These results suggest that Class I Fbas from eubacteria are structurally closer to the archaeal subfamily of Fba rather than to the eukaryotic subfamily.

### The quaternary structure of FbaB in solution

**Fig 6:**
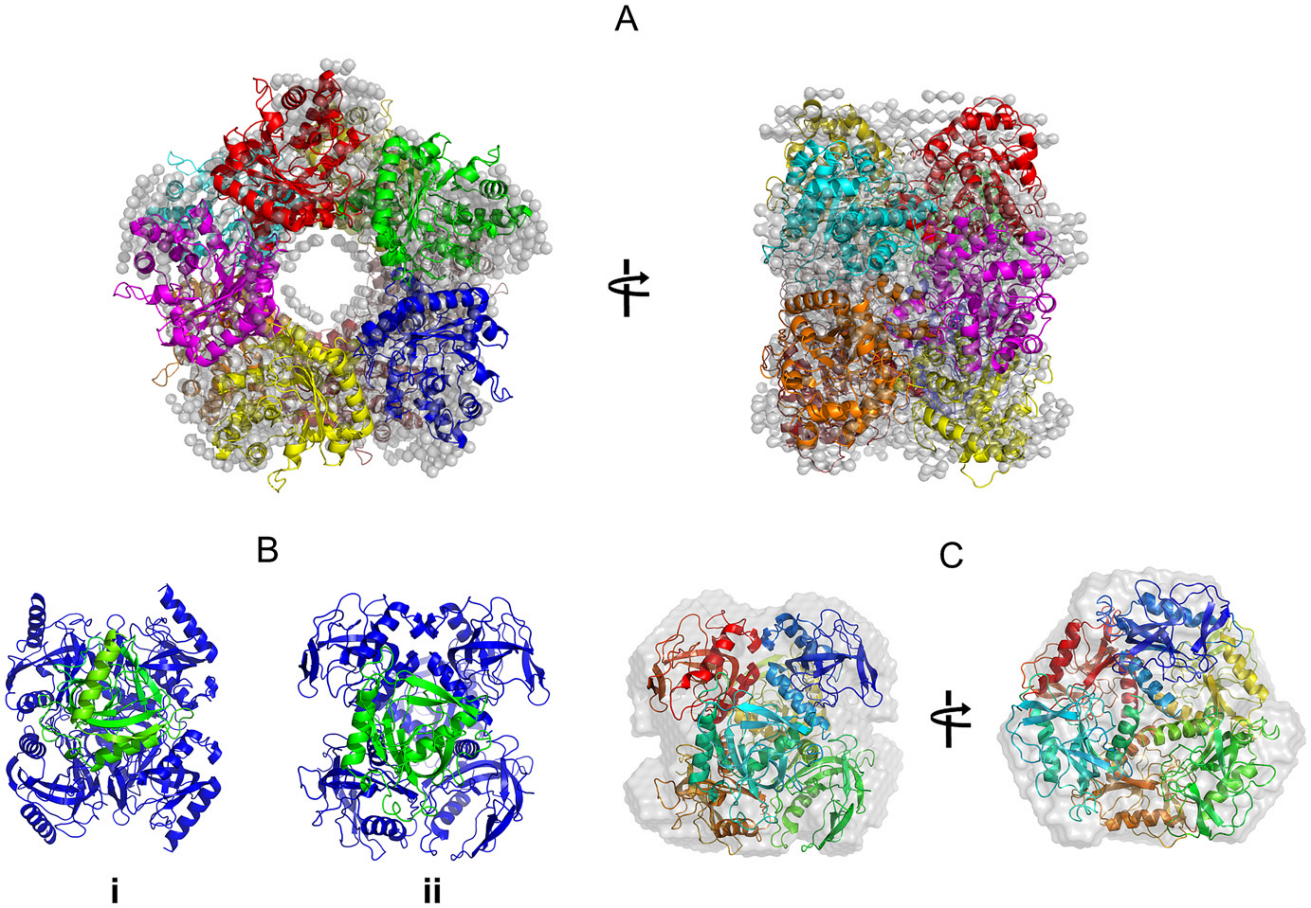
Shape restoration of FbaB and PPase by *ab initio* and rigid body methods. **A.** A DAMMIN *ab initio* model of the FbaB decamer (light grey spheres, P52 symmetry) superimposed with a rigid body model showing the arrangement of the individual protomers (represented as different colored ribbons). Two orientations are shown. **B.** A comparison of the subunit orientation of PPase from the X-ray crystal structure (i) compared to the rigid body model (ii) determined from SAXS data. A single protomer has been colored green to highlight the apparent rearrangement of the PPase subunits that occurs in solution. **C.** The DAMMIN *ab initio* bead model of the PPase hexamer (light grey spheres/surface; P32 symmetry) superimposed with the SASREF rigid-body model of the enzyme (each protomer of the PPase hexamer are shown as different colored ribbons).

An analysis of the SAXS data measured from *E*. *coli* FbaB shows that the TIM-barrel protomers of the enzyme self-associate and form a decamers in solution. The distance distribution function, *p*(*r*), of FbaB (Fig 1A, insert) yields a maximum particle dimension, *D*_*max*_, of 12.7±0.6 nm. The corresponding low resolution shapes of FbaB reconstructed by DAMMIN (based on the *p*(*r*)) provide oblate bead models (Fig 6A) that are spatially consistent with each other (average NSD in P52 symmetry = 0.8) and fit the experimental data well (*χ*^2^ = 1.2; Fig 1A, curve 2). A more detailed model of the quaternary structure was generated using the FbaB monomer generated by I-Tasser as a rigid body for SASREF and CORAL modeling. The FbaB enzyme from eukaryotes is a tetramer [3] whereas FbaB from archaea family forms decamers [53] so that in principle FbaB from *E*. *coli* might be expected to vary between tetramers and decamers. The MM of FbaB evaluated from SAXS data (Table 1) indicates that the number of subunits in the protein should be not less than eight and we attempted to construct both octameric and decameric models. Using the well-ordered TIM-barrel core of the FbaB protomer as a rigid body, an octamer model was built using SASREF in P42 symmetry which yielded a poor fit to the experimental SAXS data (*χ*^2^ = 4.8; Fig 1A, curve 3). Increasing the number of subunits to ten using P52 symmetry improved the fit of the FbaB model (*χ*^2^ = 3.7, Fig 1A, curve 4) suggesting that the protein’s quaternary structure is indeed a decamer. To further refine decameric model we used CORAL that also determined the shape and spatial disposition of the N-terminal extension to the TIM-barrel fold as shown in Fig 4 (modeled as a string of dummy atoms). The CORAL model provides a significant improvement in the fit (*χ*^2^ = 1.8, Fig 1A, curve 6) suggesting that the new type of FbaB is decameric in solution (Fig 6A). This conclusion is also supported by molecular mass estimations determined from the SAXS data. The MM_aa_ of the predicted monomer is approximately 38 kDa whereas the MM obtained from MM_*I*(0)_ and MM_Porod_ are 340kDa and 335kDa, respectively. These values are consistent with the enzyme adopting a decameric structure in solution. Of note, an archaeal Class I FbaB is also a decamer (PDB: 1OJX; *Thermoproteus tenax*) [3], however the scattering calculated from this structure does not fit the experimental *E*. *coli* FbaB SAXS data (*χ*^2^ = 10.0, Fig 1A, curve 5) indicating that the quaternary structures of the two decamers are quite different.

### Quaternary structure of PPase in solution

The overall structural parameters computed from the SAXS data by PPase are given in Table 1. The *p*(*r*) (Fig 1B, insert) is characteristic for a compact protein and the structural parameters determined from the SAXS data are in reasonable agreement with those parameters calculated from the X-ray crystallographic model of PPase (PDB: 2AUU [23]; *R*_*g*_ = 3.0 nm, *D*_*max*_ = 9.6 nm). PPase forms a compact hexamer both in solution and in the crystal matrix. However, the calculated scattering profile computed from the X-ray crystal structure using CRYSOL (Fig 1B, curve 3) does not fit the experimental data (*χ*^2^ = 5.2; Fig 1B, curve 1) indicating that differences are likely present between the crystal and overall solution conformation(s) of the protein. SASREF rigid body modeling refinement of PPase using P32 symmetry reduced the discrepancy to *χ*^2^ = 1.4 (Fig 1B, curve 4). The main difference between the crystal and solution conformations appears to be a rotational shift in the orientation of the individual PPase subunits (Fig 6B). The overall low-resolution shapes reconstructed by DAMMIN (average NSD in P32 symmetry = 0.6) fit the experimental curve very well with discrepancy *χ*^2^ = 1.0 (Fig 1B, curve 2) and are spatially superimposable with the SASREF rigid body model (Fig 6C).

### The solution states of KduI

**Fig 7:**
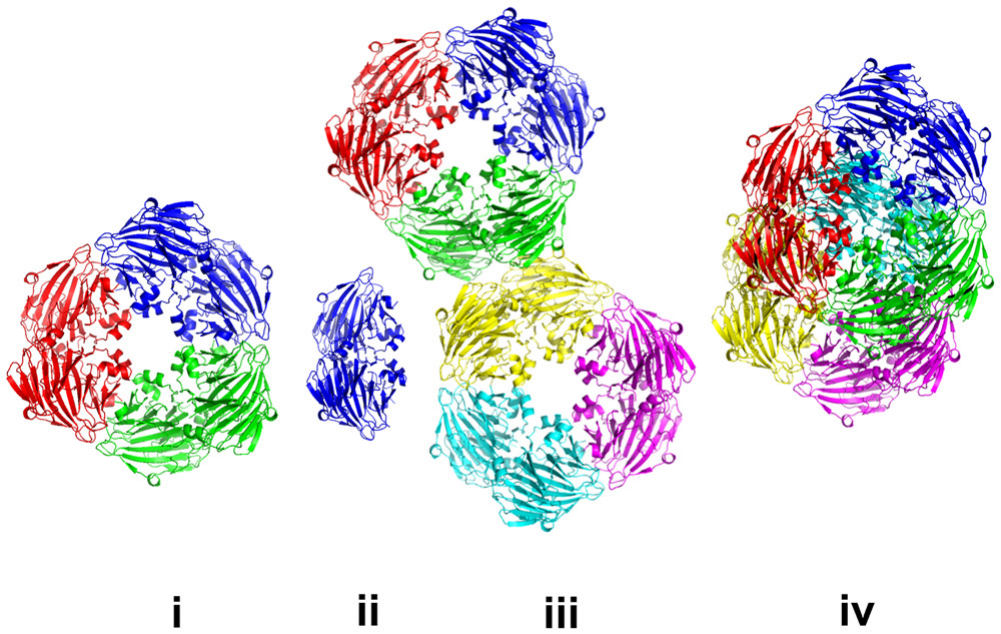
Oligomeric species of KduI. KduI consists of hexamers (i), dimers (ii), as well as extended (iii) and stacked dodecamers (iv). The corresponding fit to the SAXS data of the mixture is shown in Fig 3A, curve 3 (hexamers, *v*_*i*_ = 0.31; stacked dodecamers, *v*_*i*_ = 0.17; extended dodecamers, *v*_*i*_ = 0.24 and; dimers, *v*_*i*_ = 0.28).

From the crystal structure of *E*. *coli* KduI (PDB: 1XRU) [24] the biological unit is expected to be a homo-hexamer consisting of six 31 kDa protomers. Both size-exclusion chromatography (SEC) and analytical ultracentrifugation (AU) data show that a homo-hexamer with a sedimentation coefficient of *s*_20,w_ = 10.2 ± 0.2 S and molecular mass of 188 ± 15 kDa is the predominant form of KduI in solution (S4 Fig). However, in addition to the hexamer trace amounts of lower- and higher-mass species are also observed with apparent molecular masses of 75 ± 20 and 290 ± 20 kDa, respectively. The mass of these additional species correspond closely to the expected MM of KduI dimers and dodecamers. The SAXS data also indicate that KduI exists as a mixture of oligomeric species in solution, as initially shown by the non-linearity of the Guinier plot at low-*s*^2^ (Fig 3C). In addition, the scattering curve computed from the crystallographic hexamer alone yields a very poor agreement with the SAXS data (*χ*^2^ = 10.4; Fig 2A, curve 2). The observed differences between the X-ray crystal structure and the SAXS data, specifically at very low angles (*s* < 0.3 nm^-1^), indicate that higher molecular weight oligomers are present in the sample while systematic deviations at larger angles (*s* > 0.7 nm^-1^) suggest that the sample may also contain species that are smaller than hexamers. KduI is a member of the cupin superfamily and cupin proteins are known to form higher oligomeric states [54]. We therefore modeled the SAXS data using different possible combinations of associated and dissociated KduI oligomers using rigid-body atomistic models extracted from the X-ray crystal structure (e.g., crystallographic hexamers, dimers, and dodecamers, etc). Using OLIGOMER, the best fit to the experimental data (*χ*^2^ = 1.77; Fig 2A, curve 3) was obtained from an equilibrium mixture of hexamers (volume fraction *v*_*i*_ = 0.31), stacked dodecamers (*v*_*i*_ = 0.17), extended dodecamers (*v*_*i*_ = 0.24) and crystallographic dimers (*v*_*i*_ = 0.28). The structures of these oligomeric components are presented in Fig 7. The average MM evaluated by OLIGOMER is similar to that estimated from *I*(0) and the Porod volume obtained from the data (180 kDa; Table 1). When viewed in isolation, the MM by itself suggests KduI is hexameric. However, the OLIGOMER results demonstrate that both the experimental and modeled MM are simply coincident with the volume-fraction weighted presence of associated and dissociated forms of the protein under the solution conditions of the experiment. Note that the MM calculation is based on the forward scattering, whereas OLIGOMER utilizes the full scattering patterns allowing much more reliable assessment of the oligomeric states. The ability of KduI to form mixtures of different oligomeric species may be an important property of the protein that contributes to regulating enzymatic activity. The different oligomeric forms may differ in their catalytic efficiency, as has been established for a number of other allosteric enzymes [55].

### Solution states of GadA

According to previous investigations, the oligomeric state of GadA is pH dependent. Electron microscopy and AU equilibrium sedimentation experiments have demonstrated that at pH 4.5 GadA is hexameric, with a sedimentation coefficient *s*_20,w_ = 12.72 ± 0.35 and molecular mass of 310 kDa [56, 57]. In turn, the X-ray crystal structure of GadA obtained at pH 4.6 (PDB: 1XEY) is also hexameric [22]. Increasing the pH changes the association state of GadA. Incubation of the protein at pH 6.0 causes the dissociation of a hexameric GadA into a lower-mass form believed to be a dimer [58]. In this work we determined the oligomeric composition of GadA at pH 4.6 and 7.5 in a solution using AU and SEC. Both methods show that at pH 4.6 GadA is hexameric with molecular mass of 340 ± 50 kDa (SEC data) or 320 ± 20 kDa (AU data) and with a *s*_20,w_ = 14.0 ± 0.2 S. At pH 7.5, a dimeric form of the protein appears with *s*_20,w_ = 7 ± 1 S and molecular mass of 120 ± 20 kDa (S5 Fig). Despite forming a well-defined peak on the SEC profile, the dimer generates a wide asymmetric peak on the AU profile (S5C Fig) attesting that the dimer may be conformationally heterogeneous.

**Fig 8:**
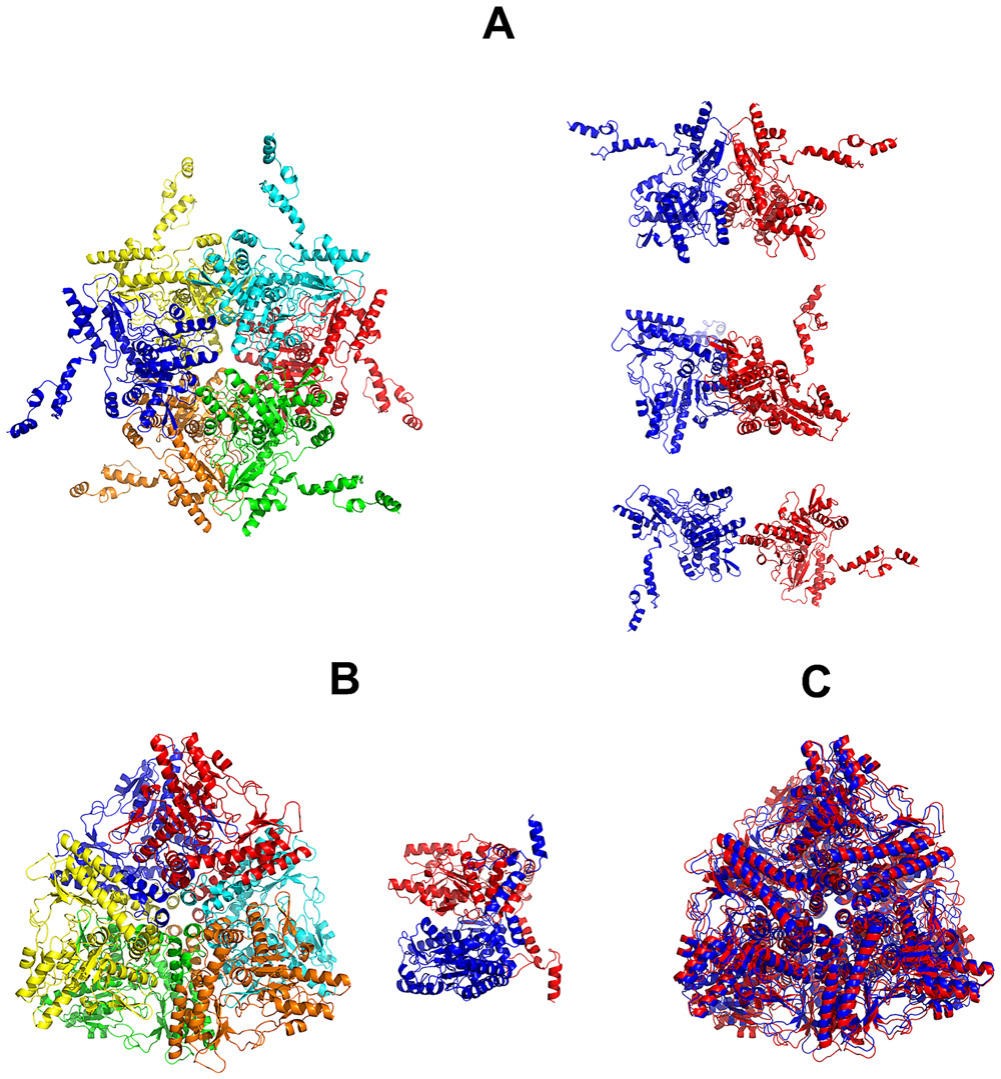
Oligomeric species of GadA. GadA consists of hexamers and dimers in solution that are structurally sensitive to sample conditions. **A.** SASREFMX models of the GadA hexamer and associated dimes from samples stored in higher ionic strength conditions. **B.** SASREFMX models of the GadA hexamer and dimer in low-salt conditions. **C.** Spatial overlay of the GadA crystal structure (red) with the GadA hexamer from the hexamer-dimer mixture obtained in low-salt conditions. The associated fits of the mixtures are presented in Fig 2B and 2C.

In agreement with the SEC and AU observations, the MM of GadA determined from the SAXS data at pH 7.5 is lower than that expected for a hexamer in both the standard buffer and in a buffer of low ionic strength (Table 1). In turn, the calculated scattering derived from the hexameric crystal structure of the biological assembly does not fit either SAXS profile, although the SAXS data obtained from the low salt sample does appear to have more features in common with the scattering calculated for the crystallographic hexamer (GadA, low-salt *χ*^2^ = 5.6; Fig 2C, curve 2) in comparison to the data measured from the samples in standard buffer (GadA, *χ*^2^ = 38; Fig 2B, curve 2). Using OLIGOMER, the scattering profiles for GadA mixtures consisting of the crystallographic hexamer and all of the associated crystallographic dimers were fitted against the experimental data (Fig 2B and 2C, curve 3). In both cases, an improvement in the fit to the data is obtained which is consistent with the observations from SEC and AUC in that GadA exists as a mixture of dimers and hexamers (GadA, *χ*^2^ = 11 and GadA low-salt *χ*^2^ = 2.0). However, the *χ*^2^ values and a visual inspection of the fits indicate that additional structural discrepancies likely exist in the hexamer-dimer mixture in solution compared to the hexamer-dimer models extracted from the X-ray crystal structure. Therefore, to further interrogate the structures of the GadA mixtures, rigid-body modelling was performed using SASREFMX of the hexamer in P32 symmetry that also included the dimeric dissociation products. The SASREFMX modeling generates hexamer-dimer mixtures with greatly improved fits to the data, especially for GadA in standard buffer (*χ*^2^ = 1.4, Fig 2B, curve 4) indicating that significant protomer rearrangements can occur in the protein at high pH and in the presence of NaCl (Fig 8A). However, under low salt conditions the improvement to the fit of the data of the hexamer-dimer mixture is less pronounced (GadA, low-salt *χ*^2^ = 1.8; Fig 2C, curve 4) suggesting that the arrangement of the GadA protomers in solution are more in keeping with the respective arrangements of the hexamers and dimers observed in the crystal structure (Fig 8B and 8C). Aside from differences in the protomer arrangement of the hexamer-dimer assemblies, the GadA sample in the low-salt condition also has a higher proportion of hexamers. OLIGOMER analysis indicates that the volume fraction of hexamer increases, from ν_i_ ≈ 0.6 to 0.8 and the dimer fraction decreases, from ν_i_ ≈ 0.4 to 0.2 as the salt concentration is decreased. The OLIGOMER results are consistent with the increased MM_*I*(0)_ estimate determined from the low-salt GadA SAXS data (Table 1), confirming that lowering the ionic strength promotes hexamer formation. Combined, the results obtained from SAXS, SEC and AU all indicate that at pH 7.5 GadA exists as dimer-hexamer mixture and at the arrangement of each GadA protomer in the GadA assemblies is likely sensitive to the sample environment. As it is known that GadA interacts with other proteins at neutral pH [31], the observed dissociation of the enzyme in combination with what appears to be an innate ability to of the hexamer to undergo structural rearrangements could be important for revealing new binding interfaces that form additional protein-protein interaction sites for modulating enzyme activity within cells.

## Conclusion

The present study is specifically focused on the first step of understanding the solution properties of four enzymes, FbaB, PPase, KduI, and GadA by elucidating the quaternary structure of each protein in solution through the application of SAXS and complementary hybrid methods. We demonstrate that all four enzymes have the ability form multimeric structures in solution. Using secondary and tertiary structure prediction in combination with experimental SAXS we demonstrate that FbaB—an enzyme with a previously unknown structure—can associate into decamers where each individual protomer likely has a core TIM-barrel fold. PPase was found to form hexamers in solution, similar to the previously determined X-ray crystal structure, while KduI and GadA, although predominantly forming hexameric assemblies exist as equilibrium mixtures that contain additional oligomeric states. KduI is best described as an equilibrium mixture of hexamers, disassociated dimers and higher-order dodecamers, whereas hexamers of GadA can rearrange and dissociate into dimers. Interestingly, for the latter protein, noticeable changes in the scattering pattern and in the oligomeric equilibrium were observed upon lowering the ionic strength of the buffer. At higher ionic strength, GadA demonstrated concentration-independent dissociation into dimers, while in a low ionic strength solution, the protein was driven closer to the hexameric form observed in the crystal structure. The effect should probably be attributed to the surface charge effects influencing the interactions between the protomers of oligomeric GadA.

The enzymes at the focus of this investigation are all thought to be involved in stress responses of bacterial cells during stationary phase, in particular glucose starvation (FbaB and possibly KduI) and growth phase-dependent acidification (GadA) [10, 21]. FbaB is present only in the cells grown on non-glucose carbon sources [59], while the products of the pathway for the utilization of non-glucose carbon sources that involves KduI (i.e., glyceraldehyde-3-phosphate and pyruvate) may be used in the second pathway involving FbaB (gluconeogenesis) [31]. GadA is localized in the cytoplasm at neutral pH, but it is found near inner membrane when the pH falls. The pH-dependent conformational change of GadA is probably related to glutamate-dependent acid resistance system [21]. PPase activity producing P_i_ may be indirectly coupled with the transmembrane transport systems [60]. Another possible role of PPase in complex with other proteins may be stabilization these proteins against unfolding and the consequent proteolytic degradation [31]. Thus, all of the studied proteins are multifunctional and their structures, in particular those that undergo association/disassociation, may speak to how cells manifest multiple molecular responses for adaption to cellular stress under the limited resources available during stationary phase. Unfortunately, attempts to demonstrate that complexes form between PPase and FbaB or with KduI and GadA under the solution conditions used for SAXS were unsuccessful (S6 Fig) even though the formation of dynamic complexes between these proteins has been demonstrated *in vitro* using pull-down assay [31]. The analysis of the solution structure(s) of the individual proteins reported here provides better understanding of their possible function in the cell. In particular, the equilibrium between oligomeric forms observed for KduI and GadA may be affected by their protein partners, thus enabling the basis for regulation of their activity.

## Supporting Information

S1 Fig Experimental SAXS patterns from FbaB (A), PPase (B), KduI (C) and GadA (D) at concentrations in the range of 1.4–10.8 mg/ml. Concentration effects on the SAXS data appear minimal. When normalized to protein concentration the SAXS data for each respective protein spanning the concentration series are superimposable. The plots show SAXS data collected at sample concentrations of 1: 1.4; 2: 5.2; 3: 8.3 and; 4: 10.8 mg/ml.
(TIF)

S2 Fig SAXS profile overlay comparing GadA (black) and GadA low-salt (red) samples. The difference between the two samples is the addition of 10 mM NaCl to the storage buffer of the GadA variant. Data have been scaled to low-*s* (*s* < 0.5 nm^-1^).
(TIF)

S3 Fig Predicted secondary structure of FbaB. α-Helices are shown as cylinders, β-strands as arrows and coils as a thick line. The color scheme of the secondary structure elements is the same as shown in Fig 1 of the main text. The confidence of the secondary structure prediction is plotted below each amino acid of the primary sequence; the confidence scale is 0–9 where 9 is the highest confidence that a predicted secondary structure element maps to the corresponding amino acid).
(TIF)

S4 Fig Size-exclusion chromatography and analytical centrifugation profiles of KduI. Panel A: SEC elution trace of KduI showing the majority of the sample consists of hexamers (peak 1) with additional lower concentrations of dodecamers (peak 2) and dimers (peak 3). Panel B: AU sedimentation profiles of KduI in solution showing the sedimentation coefficients of KduI hexamers (peak 1), dodecamers (peak 2) and dimers (peak 3).
(TIF)

S5 Fig Size-exclusion chromatography (A, B) and analytical centrifugation (C) profiles of GadA. Protein was pre-incubated at pH 4.6 (A) or 7.5 (B, C). Panel A: SEC elution trace of GadA at pH 4.6 where peak 1 corresponds to GadA hexamers. Panel B: SEC elution trace of GadA at pH 7.5 showing that on increasing the pH a GadA dimer peak appears. Panel C: AU sedimentation profiles of GadA showing the sedimentation coefficient of GadA hexamers (peak 1) and dimers (peak 2).
(TIF)

S6 Fig Scattering data from PPase/FbaB, PPase/GadA and PPase/KduI mixtures. In all panels profile 1 displays the experimental SAXS data measured from the combined/mixed samples of: A, PPase/FbaB; B, PPase/GadA; C, PPase/KduI. Curve 2 in each panel shows the scattering patterns computed using a volume-fraction weighted sum of the experimental SAXS data recorded from the purified individual components prior to mixing. It is possible to describe the SAXS data of the mixtures as PPase + FbaB, or PPase + GadA, or PPase + KduI, respectively, using the following volume fraction estimates: (A) PPase: *v*_*i*_ = 0.14, FbaB; *v*_*i*_ = 0.86; (B) PPase: *v*_*i*_ = 0.23, GadA: *v*_*i*_ = 0.77; (C) PPase: *v*_*i*_ = 0.42, KduI *v*_*i*_ = 0.58. These results show that each sample is a simple mixture and that PPase does not readily form a complex or bind to FbaB, GadA or KduI under the experimental conditions used for SAXS.
(TIF)

S1 Table Top 10 structural analogs of FbaB model identified by I-Tasser in PDB. (DOCX)
