# Supplementary figures and images for: X-Ray Solution Scattering Study of Four Escherichia coli Enzymes Involved in Stationary-Phase Metabolism
Source: PLoS One. 2016 May 26;11(5):e0156105. doi: 10.1371/journal.pone.0156105 (PMC4881948; doi:10.1371/journal.pone.0156105)

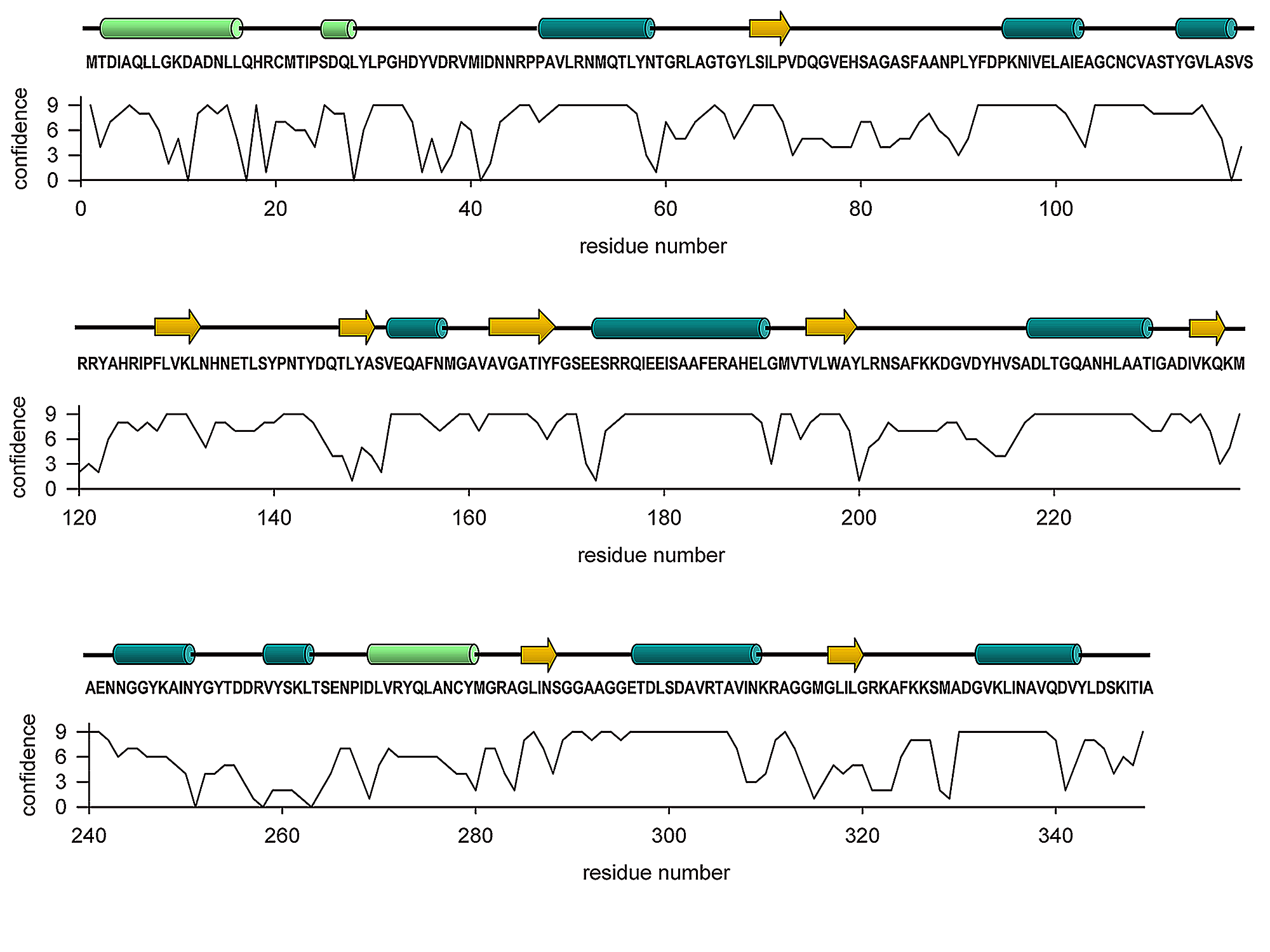

Supplement: S3 Fig — α-Helices are shown as cylinders, β-strands as arrows and coils as a thick line. The color scheme of the secondary structure elements is the same as shown in Fig 1 of the main text. The confidence of the secondary structure prediction is plotted below each amino acid of the primary sequence; the confidence scale is 0–9 where 9 is the highest confidence that a predicted secondary structure element maps to the corresponding amino acid). (TIF) [file pone.0156105.s003.tif]
