# Supplementary material for: X-Ray Solution Scattering Study of Four Escherichia coli Enzymes Involved in Stationary-Phase Metabolism
Source: PLoS One. 2016 May 26;11(5):e0156105. doi: 10.1371/journal.pone.0156105 (PMC4881948; doi:10.1371/journal.pone.0156105)
Supplement: S1 Table — (DOCX) [file pone.0156105.s007.docx]

S1 Table. Top 10 structural analogs of FbaB model identified by I-Tasser in PDB.

| Rank | pdb ID, chain | Protein | TM-score^1^ | Rmsd, Å | Coverage^2^ | Ref. |
| --- | --- | --- | --- | --- | --- | --- |
| 1 | 4mozA | Fructose-1,6-bisphosphate aldolase | 0.758 | 2.22 | 0.814 | tbp^3^ |
| 2 | 2qjgA | 2-amino-3,7-dideoxy-D-threo-hept-6-ulosonic acid synthase (Class I aldolase) | 0.720 | 1.69 | 0.757 | [S1] |
| 3 | 3mhgA | Tagatose-1,6-bisphosphate aldolase | 0.696 | 2.70 | 0.777 | [S2] |
| 4 | 3myoA | Tagatose-1,6-bisphosphate aldolase | 0.693 | 2.72 | 0.777 | [S3] |
| 5 | 3iv3A | Putative tagatose-1,6-bisphosphate aldolase | 0.688 | 2.78 | 0.774 | tbp |
| 6 | 3kaoA | Tagatose-1,6-bisphosphate aldolase | 0.688 | 2.74 | 0.771 | tbp |
| 7 | 3gndC | LsrF, function unidentified, putative Class I aldolase | 0.680 | 2.08 | 0.731 | [S4] |
| 8 | 1ok6A | Fructose-1,6-bisphosphate aldolase | 0.678 | 1.94 | 0.723 | [S5] |
| 9 | 3mmtA | Fructose-1,6-bisphosphate aldolase | 0.656 | 3.77 | 0.800 | [S6] |
| 10 | 3bv4A | Fructose-1,6-bisphosphate aldolase | 0.655 | 3.78 | 0.794 | [S7] |

^1^TM-score of the structural alignment between the query structure and structural analog from pdb.

^2^Coverage of the structure alignment by TM-align equal to the number of structurally aligned residues divided by length of the query protein.

^3^Structure announced as “to be published” in pdb.

References:

S1. Morar M, White RH, Ealick SE. Structure of 2-amino-3,7-dideoxy-D-threo-hept-6-ulosonic acid synthase, a catalyst in the archaeal pathway for the biosynthesis of aromatic amino acids. Biochemistry. 2007; 46: 10562-10571.

S2. LowKam C,  Liotard B,  Sygusch J. Structure of a class I tagatose-1,6-bisphosphate aldolase: investigation into an apparent loss of stereospecificity. J.Biol.Chem. 2010; 285: 21143-21152.

S3. Lee SJ,  Kim HS,  Kim DJ,  Yoon HJ,  Kim KH,  Yoon JY, et al. Crystal structures of LacD from Staphylococcus aureus and LacD.1 from Streptococcus pyogenes: Insights into substrate specificity and virulence gene regulation. Febs Lett. 2011; 585: 307-312.

S4. Diaz Z, Xavier KB, Miller ST. The crystal structure of the Escherichia coli autoinducer-2 processing protein LsrF. Plos One. 2009; 4: e6820-e6820.

S5. Lorentzen E, Pohl E,  Zwart P, Stark A, Russell R, Knura T, et al. Crystal Structure of an Archaeal Class I Aldolase and the Evolution of (Beta Alpha)8 Barrel Proteins. J.Biol.Chem. 2003; 278: 47253-47260.

S6. Gardberg A, Abendroth J, Bhandari J, Sankaran B, Staker B. Structure of fructose bisphosphate aldolase from Bartonella henselae bound to fructose 1,6-bisphosphate. Acta Crystallogr.,Sect.F. 2011; 67: 1051-1054.

S7. Sherawat M, Tolan DR,  Allen KN. Structure of a rabbit muscle fructose-1,6-bisphosphate aldolase A dimer variant. Acta Crystallogr.,Sect.D.2008; 64: 543-550.
